# Management and cost of snakebite injuries at a teaching and referral hospital in Western Kenya

**DOI:** 10.12688/f1000research.20268.1

**Published:** 2019-09-04

**Authors:** Mitchel Otieno Okumu, Minal Naran Patel, Foram Rajnkant Bhogayata, Francis Okumu Ochola, Irene Awuor Olweny, Joshua Orungo Onono, Joseph Kangangi Gikunju

**Affiliations:** 1 Pharmacy, Jaramogi Oginga Odinga Teaching and Referral Hospital, Kisumu, Kenya; 2 Public Health, Phamacology and Toxicology, Faculty of Veterinary Medicine, University of Nairobi, Nairobi, Kenya; 3 Pharmacology and Toxicology, Moi University, Eldoret, Kenya; 4 Medical Laboratory Science, Jomo Kenyatta University of Agriculture and Technology, Nairobi, Kenya

**Keywords:** snakebite, snakebite envenoming, costs of snakebite, universal health coverage, neglected tropical disease, snakebite burden, sub-Saharan Africa, management of snakebite

## Abstract

**Background: **Data on the cost of snakebite injuries may inform key pillars of universal health coverage including proper planning, allocation, and utility of resources. This study evaluated the injuries, management, and costs resulting from snakebites at Jaramogi Oginga Odinga Teaching and Referral Hospital (JOOTRH) in Kenya.

**Methods: **In total, medical records of 127 snakebite victims attending JOOTRH between January 2011 and December 2016 were purposely selected and data on the age, gender, type of residence (urban or rural), part of the body bitten, time of bite, injuries, pre-hospital first aid, time to hospital, length of stay, treatment, and costs were collected. Regression analysis was used to predict the total indirect cost of snakebite injuries and *p≤ 0.05 *was considered significant. Mortality and loss of income of hospitalized victims were considered as direct costs.

**Results: **It was found that 43 victims were 13-24 years of age, 64 were female, 94 were from rural areas, 92 were bitten on the lower limbs, 49 were bitten between 6.00 pm and midnight, 43 attempted pre-hospital first aid, and the median time to hospital was 4.5 hours. Antivenom, supportive therapy, antibiotics, antihistamines, corticosteroids, analgesics, and non-steroidal anti-inflammatory drugs were used. Cellulitis, compartment syndrome, gangrenous foot, psychiatric disorder, and death were the main complications. Most victims spent 1-5 days in hospital and the median cost of treating a snakebite was 2652 KES (~$26). Drugs, ward charges, and nursing procedures were the highest contributors to the total indirect cost. Victims hospitalized for 6-10 days and >10 days incurred 32% and 62% more costs, respectively, compared to those hospitalized for 1-5 days.

**Conclusions: **The longer snakebite victims are hospitalized, the higher the cost incurred. Continuous medical education on the correct management of snakebites should be encouraged to minimize complications that may increase hospital stays and costs incurred.

## Introduction

Snakebite envenoming is a topic perhaps too little discussed on the global stage. The disease has only recently been formally re-instated to the WHO list of neglected tropical diseases after a four-year hiatus ^1^. The additional focus is welcome and has been widely anticipated by many people that deal with and understand the devastating impact of snakebite envenoming on individuals, their families, and communities in general. It is widely hoped that the reinstatement will significantly boost efforts to reduce the burden of snakebite in Asia, Africa, and Latin America where the burden is highest ^1, 2^.

Specifying the requirements for antivenom at the local level, educating at-risk groups, improving accessibility to antivenoms, and training of healthcare personnel are the major challenges in addressing snakebites ^1^. Most of the mitigative strategies focus on ways and means of addressing these challenges ^1^. However, evaluating the cost associated with managing snakebites at the local hospital level has often been overlooked. To date, there are no studies that provide estimates for how much it may cost to treat a victim of a snakebite at the local hospital setting.

Kenya is a country with a population of about 40 million people ^3^. Access to affordable healthcare in the country has often been fraught with controversy ^4^. In a bid to provide quality healthcare to its citizens, the government of Kenya has recently adopted the big four agenda ^5^, which seeks to deliver healthcare, food security, manufacturing growth, and affordable housing ^5^. To deliver on healthcare, the government has embraced the Kenyan Health Policy, which borrows heavily from the WHO model on Universal Healthcare Coverage (UHC) ^6^. The aim is to ensure that every Kenyan receives quality healthcare (promotive, preventive, curative and rehabilitative) without suffering financial strain ^7^. 

There has been a huge clamor for UHC in Kenya. However, only four counties/administrative units, namely Kisumu, Isiolo, Nyeri, and Machakos, have been selected to pilot the rollout of the program ^7^. Data on the cost of treating various diseases may inform key pillars of UHC, including proper health planning, resource allocation and utility of resources. Snakebite injuries are probably one of the most hidden public health crises globally. However, in a country that is grappling with HIV/AIDS, malaria, tuberculosis and other non-communicable diseases ^8^, there is a real risk that snakebites may be further neglected in terms of policy and resource allocation. Estimating the cost of managing snakebite injuries at the local hospital level may go a long way in highlighting the gaps that require the attention of the government and other relevant stakeholders.

In a previous study on acute poisoning at Jaramogi Oginga Odinga Teaching and Referral Hospital (JOOTRH), we established that snakebites were the leading cause of poisoning between the years 2011 and 2016 ^9^. Therefore, the objective of the present study was to evaluate the injuries, management, and costs associated with snakebites at the hospital between the years 2011 and 2016. The information may be important for guiding policy on proper health planning, resource allocation and utility of the limited resources available to the counties.

## Methods

### Ethical considerations

Permission to conduct the study was obtained from the hospital administration; ethical clearance was obtained from the Ethical Review Committee (ERC) of JOOTRH (Ref no: ERC.IB/VOL1/412). The ethical approval document is provided as *Extended data* (Figure S1) ^10^.

### Study design

This was a descriptive cross-sectional study that was carried out between July and November 2017 using data from a retrospective audit on records of acute poisoning at JOOTRH ^9^. All patients with acute poisoning due to snakebite envenoming presenting to and managed in the emergency department of JOOTRH between January 2011 and December 2016 were reviewed for inclusion. Victims transferred from health centers, dispensaries, and sub-county hospitals to JOOTRH were counted once after thorough scrutiny of the medical records to ensure there was no double reporting. The medical records had to have all the study variables of interest; age, gender, type of residence (urban or rural), part of the body bitten, time of bite, injuries, pre-hospital first aid, time to hospital, length of stay, treatment, and costs. Any medical records that had incomplete information or did not have any of these parameters of interest were excluded from the analysis. In total, 127 medical records of snakebite victims attending the hospital between January 2011 and December 2016 were purposely selected for the study. The digital archiving of medical records at JOOTRH based on the international classification of diseases begun in January 2011 and this made it easier for us to access/trace the physical medical records, as opposed to periods before the digital archiving was in place. We therefore selected a five-year time frame from the period of inception of the digital archiving as our study period. Data on the age, gender, type of residence (urban or rural) of the victim, part of the body bitten, and the time of the bite was retrieved. Other data retrieved included the type of injuries, pre-hospital management measures, time taken to reach the hospital, length of stay at the hospital, type of treatment, and associated costs. Indirect costs included those related to registration, admission, file fees, laboratory, laundry, physiotherapy, nursing procedures, antivenom, other drugs, daily ward charges, caretaker costs, cost of non-pharmaceuticals, surgical operations on victims, and miscellaneous. The direct costs considered in this analysis were those incurred due to mortalities and loss of income by patients who were hospitalized.

### Study setting

The study was carried out at JOOTRH, a referral hospital in Kisumu County. In December of 2018, the county was selected as the first of four counties to pilot UHC in Kenya ^7^. The hospital has a wide catchment area encompassing up to 10 counties (Kisumu, Siaya, Homa Bay, Migori, Kisii, Kakamega, Vihiga, Bungoma, Busia, and Nandi) within the Western Kenya region. Based on estimates from the most recent census (2009), these counties are estimated to have a population of around 970, 000, 840,000, 750,000, 260,000, 1,150,000, 1,660,000, 550, 000, 1,600,000, 490, 000, and 750,000 people, respectively ^9^. Curative, preventive, promotive and rehabilitative health services are provided at JOOTRH ^9^.

### Data handling and statistical analysis

Sociodemographic data from the pre-structured proforma were collated in MS Excel spreadsheets and analyzed using descriptive statistics. Categorical variables were presented in frequencies and percentages and qualitative variables were handled through thematic analysis. Quantitative data including additional costs of treatment were analyzed using descriptive statistical measures including measures of central tendency; mean, median and measures of dispersion: minimum, and maximum. Multiple linear regression was used to determine the predictors of the total indirect cost of treating snakebite (SPSS version 20.0). The total indirect cost (dependent variable) was transformed to log _10_ and regressed against independent variables including the age, gender, and type of residence (urban or rural) of victims, location of the victims at the time of the bite, season and time of the bite, and the time taken to get to the hospital. Other independent variables were the length of hospital stay, first aid measures initiated, and part of the body bitten. *p<0.05* was considered significant. The direct costs considered in this analysis were those incurred due to mortalities and loss of income by patients who were hospitalized.

## Results

The year of the bite, the type of residence (urban or rural) of victims and the seasonal distribution of snakebite in the catchment area of the hospital is summarized in Table 1 ^11^. Most bites occurred in the year 2012, with Kisumu County recording the highest number of bites. Moreover, most bites took place during the rainy season.

**Table 1.** Year of bite, county of origin, and seasonal distribution of the snakebite victims who presented to Jaramogi Oginga Odinga Teaching and Referral Hospital during the study period.

Variable	Frequency *(n=127)*
**Year**	
2011	19 (14.9%)
2012	30 (23.6%)
2013	24 (18.9%)
2014	27 (21.3%)
2015	18 (14.2%)
2016	9 (7.1%)
**County**	
Kisumu	101 (79.5%)
Siaya	13 (10.2%)
Vihiga	5 (3.9%)
Homabay	3 (2.4%)
Nandi	3 (2.4%)
Kakamega	1 (0.8%)
Migori	1 (0.8%)
**Season**	
Long rains 1 (March-May)	26 (20.5%)
Long rains 2 (Oct-Nov)	25 (19.7%)
Short rains (Aug-Sep)	31 (24.4%)
Cool dry season (Jun-July)	25 (19.7%)
Hot dry season (Dec-Feb)	20 (15.7%)

The majority of the victims (110/127, 86.6%) presented to hospitals within Kisumu County, 8/127 (6.3%) cases presented to hospitals in Siaya County, 4/127 (3.1%) cases presented to hospitals in Vihiga County, 2/127 (1.6%) cases presented to hospitals in Homabay County, one (0.8%) case presented to a hospital in Busia County and another one (0.8%) case presented to a hospital in Migori County. Of the 127 cases, 84 (66.1%) presented to JOOTRH as the first port of call. This was followed by nine cases (7.1%) who presented to Ahero Sub-County hospital, four cases (3.1%) who presented to the Kombewa Sub-County hospital, and another four cases (3.1%) who presented to the Nyakach Sub-County hospital (Table S1 *, Extended data*) ^10^. The gender, type of residence, marital status, and age of the victims who presented to JOOTRH during the study period are summarized in Table 2. About 94/127 (74.0%) of all snakebite cases were from the rural areas and most victims were between 13 and 24 years of age.

**Table 2.** Gender, type of residence, marital status, and age of snakebite victims who presented to Jaramogi Oginga Odinga Teaching and Referral Hospital during the study period.

Variable	Frequency *(n=127)*
**Gender**	
Male	63 (49.6%)
Female	64 (50.4%)
**Type of residence**	
Rural	94 (74.0%)
Urban	33 (26.0%)
**Marital status**	
Single	31 (24.4%)
Married	48 (37.8%)
Divorced	1 (0.8%)
Widowed	3 (2.4%)
Child	35 (27.6%)
Not captured	9 (7.1%)
**Age (years)**	
0-12	31 (24.4%)
13-24	43 (33.9%)
25-36	25 (19.7%)
37-48	13 (10.2%)
49-60	9 (7.1%)
61-72	4 (3.1%)
73-84	2(1.6%)

The occupation of snakebite victims is summarized in Table 3.

**Table 3.** Occupation of the victims of snakebite who presented to Jaramogi Oginga Odinga Teaching and Referral Hospital during the study period.

Variable	Frequency *(n=127)*
**Occupation**	
Attending school	47 (37.0%)
Entrepreneur	20 (15.7%)
Farming	18 (14.2%)
Toddler	8 (6.3%)
Not captured	7 (5.5%)
Laborer	5 (3.9%)
Unemployed	5 (3.9%)
Artisan	5 (3.9%)
Homemaking	4 (3.1%)
Security	3 (2.4%)
Hospitality	3 (2.4%)
Teaching	2 (1.6%)

The site of the bite, location of the victim at the time of the bite, and the circumstance/activity during which the snakebite occurred are shown in Table 4. Most bites were on the lower limbs, occurred outdoors, and took place while the victims were walking.

**Table 4.** Site of the bite, location, and activity of the victim at the time of the bite.

Variable	Frequency *(n=127)*
**Site of bite**	
Upper limbs	20 (15.7%)
Lower limbs	92 (72.4%)
Other	2 (1.6%)
Not specified	13 (10.2%)
**Location of the victim at** **the time of the bite**	
Indoors	15 (11.8%)
Outdoors	71 (55.9%)
Unspecified	41 (32.3%)
**Circumstance/activity**	
Walking	54 (42.5%)
Unknown	31 (24.4%)
Resting/sleeping	12 (9.4%)
Farming	12 (9.4%)
Working	10 (7.9%)
Playing	6 (4.7%)
Relieving him/herself	2 (1.6%)

Most bites occurred between 1800 and 2359 and a majority of the victims did not attempt any form of pre-hospital measure after being bitten ( Table 5).

**Table 5.** Time of bite, and pre-hospital first aid measures taken by victims who presented to Jaramogi Oginga Odinga Teaching and Referral Hospital during the study period.

Variable	Frequency *(n=127)*
**Time of bite**	
06:00–11:59	22 (17.3%)
12:00–17:59	24 (18.9%)
18:00–23:59	49 (38.6%)
00:00–05:59	9 (7.1%)
No data	23 (18.1%)
**Pre-hospital measures**	
None	86 (67.7%)
Tourniquet only	14 (11.0%)
Herbal medicine only	10 (7.9%)
Tourniquet, incisions, herbal medicine	9 (7.1%)
Herbal medicine, incisions	3 (2.4%)
Incisions only	2 (1.6%)
Burning matchstick at the site of the bite	1 (0.8%)
Herbal medicine, limb immobilization	1 (0.8%)
Herbal medicine, cleaning wound with cold water	1 (0.8%)
Tourniquet, cloth impregnated with charcoal	1 (0.8%)
Tourniquet, incisions	1 (0.8%)
Tourniquet, application of vaseline at the site of the bite	1 (0.8%)
Cleaning the wound with potassium permanganate	1 (0.8%)
Cleaning the wound with povidone- iodine	1 (0.8%)
Tourniquet, pouring paraffin on the site of the bite	1 (0.8%)

Most of the victims took less than six hours to present to the hospital (median time was 4.5 hours). A majority reported having been bitten by what they described as a black snake and cellulitis was the most common complication ( Table 6).

**Table 6.** The time taken to get to the hospital, description of offending snakes, and the complications of snakebite among victims who presented to Jaramogi Oginga Odinga Teaching and Referral Hospital during the study period.

Variable	Frequency
**Time taken to get to the** **hospital**	***(n=127)***
0–6 hours	54 (42.5%)
6–12 hours	14 (11.0%)
>12 hours	10 (7.9%)
No data	49 (38.6%)
**Description of offending** **snakes**	***(n=127)***
Black snake	49 (38.6%)
Snake not seen	44 (34.6%)
Green snake	13 (10.2%)
Brown snake	8 (6.3%)
Brown and black snake	7 (5.5%)
Grey snake with white spots	1 (0.8%)
Red and brown spots	1 (0.8%)
White and brown snake	1 (0.8%)
White, brown, and black snake	1 (0.8%)
White-bellied snake	1 (0.8%)
Yellow snake	1 (0.8%)
**Complications of snakebite**	***(n=30)***
Cellulitis	18 (69.2%)
Death	4 (15.4%)
Compartment syndrome	4 (15.4%)
Gangrene	2 (7.7%)
Cellulitis and gangrene	1 (3.8%)
Psychiatric episode	1 (3.8%)

The symptoms of victims of snakebite who presented to JOOTRH during the study period are summarized in Table 7. The symptoms were local and systemic (neurological, hematological).

**Table 7.** Signs and symptoms of snakebite among victims in the study area during the study period.

Classification of the symptoms	Manifestations
**Local**	Septicemia, tissue necrosis, gangrene, swelling, edematous swelling, edema, mild tenderness on palpation, tenderness, gradual/progressive swelling, leg ulcer, purulent discharge, pitting edema, cellulitis, shiny appearance of foot, bullae on leg, blistering (ruptured and unruptured), multiple bruising on lower limbs, tense skin with bullous eruptions, bland blistering, cold leg, fang marks with/without pruritus
**Systemic** **(Neurological)**	Radiating/pulsating/localized/sudden onset pain, pulse awareness, paresthesia, dysphagia, numbness, blurred vision, headache, frothing at the mouth, cough, unconsciousness, pin-point pupil, dizziness, elevated body temperature, weakness, dyspnea, loss of hearing on left ear, profuse sweating, slurred speech, easy fatiguability, nausea, vomiting
**Systemic** **(hematological)**	Slight/mild/minimal bleeding, severe/excessive bleeding, hyperpigmentation at the site of the bite, ecchymosis, erythema

The treatment received by the victims of snakebite included the use of antivenom, supportive therapy, antimicrobial agents, antihistamines, corticosteroids, non-steroidal anti-inflammatory agents, opioid and non-opioid analgesics and general anesthetics ( Table 8).

**Table 8.** An overview of the treatment regimen used for the management of snakebite in the study area.

Treatment	Description
**Antivenom**	Unspecified brands
**Supportive therapy**	Normal saline, normal saline and ringer’s lactate, glucose infusion
**Antimicrobial agents**	Floxapen IV, ceftriaxone IV, floxapen PO, metronidazole IV, benzylpenicillin IV, fluconazole PO, ampliclox PO, amoxil PO, augmentin PO, co-trimoxazole PO, gentamycin IV, metronidazole PO, cefuroxime PO, ceftazidime IV, Lincomycin IV
**Antihistamines**	Piriton IV, piriton PO, cetirizine PO
**Corticosteroids**	Hydrocortisone IV, hydrocortisone ointment, dexamethasone IV, prednisolone PO
**Non-steroidal anti-** **inflammatory drugs** **(NSAID’s)**	Diclofenac IM, ibuprofen PO, diclofenac PO, ketorolac tromethamine PO, aspirin PO, flamchek PO (chlorzoxazone:diclofenac:paracetamol:250mg/50mg/325mg), betapyn PO(paracetamol:codeine phosphate:caffeine: doxylamine succinate:450/10/50/5), aceclofenac PO
**Analgesics**	Non-opioid (paracetamol PO), opioid (morphine PO, betapyn PO (paracetamol:codeine phosphate:caffeine: doxylamine succinate:450/10/50/5), tramadol IV, tramadol PO, tramadol IM, flamchek(chlorzoxazone:diclofenac:paracetamol:250mg/50mg/325mg)
**General anesthetics**	Suxamethonium IV, bupivacaine IM, sodium thiopental IV, pancuronium bromide IV, neostigmine IV, atropine IV, halothane IV
**Miscellaneous**	Lasix IV, Mupirocin ointment (Bactroban), phytomenadione IV, lignocaine IM, Aloha PO, anti-tetanus IM, emanzen forte PO, omeprazole PO, X-tone PO, adrenaline SC, orofer PO, haloperidol PO, artane PO, aldactone PO, nifedipine PO, heavy bupivacaine IM

*IV: intravenous, PO: per oral, IM: intramuscular.*

Of the 127 victims of snakebite, 53/127 (42%) received antivenom while 74/127 (58%) did not. Of those that received antivenom, 51 survived while two died. Among the victims who received antivenom, 44 received one vial of antivenom, six received two vials of antivenom, one received three vials of antivenom, and another two received five vials of antivenom (Table S2, *Extended data*) ^10^. Among those who did not receive antivenom, 72 survived and two died.

Of the 52 victims of snakebite who survived after receiving antivenom, 15 had complications including cellulitis (n=7), compartment syndrome (n=4), gangrenous foot (n=2), a psychiatric episode (n=1), and soft tissue injury (n=1). None of the 74 victims who did not receive antivenom developed any complications.

**Figure 1.**
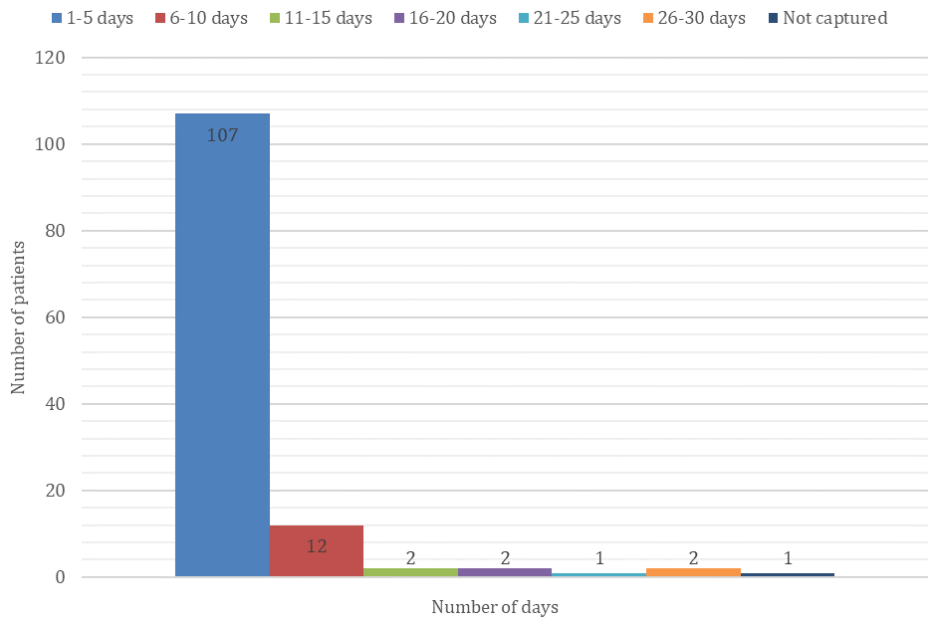
Number of days spent in the hospital by victims of snakebite in the study area.

The majority (107/127, 84%) of all victims of snakebite spent between one and five days in the hospital, 12 (9%) spent between six and 10 days, two (1.6%) spent between 11 and 15 days and another two (1.6%) spent between 16 and 20 days ( Figure 1). There was one victim (0.8%) who spent between 21 and 25 days in the hospital, while another two (1.6%) victims spent between 26 and 30 days in the hospital ( Figure 1).

When considered in terms of the daily minimum wage in Kenya (KES 452; ~ $4 USD), victims of snakebite who spent at least five, 10 and 20 days in the hospital lost about KES 2260 (~$22), KES 4520 (~$44) and KES 9, 040 (~$88) worth of wages, respectively.

**Figure 2.**
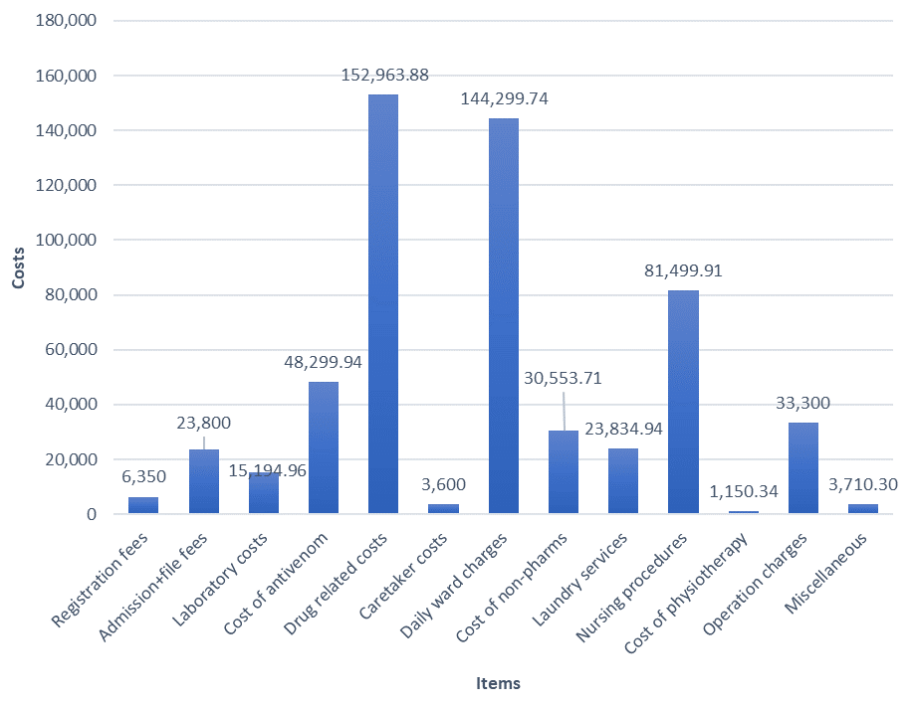
Summary of the cost of snakebite injuries in the study area.

The total indirect cost of managing snakebite in the study area during the period of study was KES 568,557.72 (~$5530). Seven victims of snakebite received a waiver on the total indirect costs amounting to 91,601 KES (~$890). None of these victims had any hospital insurance cover (Table S3, *Extended data*) ^10^. The highest contributors to the total indirect cost of snakebite were drugs (KES 152,964; ~$1485), ward charges (KES 142,300 KES; ~$1380), and nursing procedures (KES 81,500; ~$790) ( Figure 2).

The median cost of treating snakebite was KES 2652 (~$26; range: KES 1100-41399 or ~11-400$). The predictors of the total indirect cost of treating snakebite are summarized in Table 9. Generally, victims who spent 6–10 days and >10 days incurred 32% and 62% more costs, respectively, compared to those who spent 1–5 days.

**Table 9.** Overview of snakebite related factors influencing the total indirect cost of snakebite at Jaramogi Oginga Odinga Teaching and Referral Hospital.

Parameter	Estimate	Std. error	t(106)	t pr.	Odds
**Type of residence**					
Rural	-0.04	0.06	-0.64	0.52	0.96
**First aid measure**					
Did not attempt	-0.07	0.05	-1.21	0.23	0.94
**Gender**					
Female	0.03	0.05	0.54	0.59	1.03
**Location of victim**					
Outdoors ****	-0.09	0.09	-0.95	0.35	0.92
Unspecified	-0.19	1.00	-1.88	0.06	0.83
**Length of stay**					
6–10 days	0.28	0.09	3.24	0.002	1.32
>10 days	0.48	0.11	4.42	<0.01	1.00
**Part bitten**					
Upper limbs	0.00	0.07	0.05	0.96	1.00
Other	-0.19	0.20	-0.95	0.35	0.83
Unspecified	0.04	0.09	0.45	0.66	1.04
**Season**					
Wet	-0.10	0.06	-1.73	0.09	0.91
**Time of bite**					
1200–1759	-0.07	0.08	-0.91	0.37	0.93
1800–2359	0.05	0.07	0.73	0.47	1.05
0000–0559	0.16	0.13	1.26	0.21	1.17
No data	0.02	0.10	0.26	0.80	1.03
**Time to hospital**					
60–239 minutes	-0.01	0.21	-0.02	0.98	1.00
240–480 minutes	0.13	0.21	0.61	0.54	1.14
>480 minutes	-0.06	0.22	-0.28	0.78	0.94
Not captured	0.02	0.21	0.09	0.93	1.02

## Discussion

To the best of our knowledge, this is the first study that has reported on the cost of snakebites in any hospital setting. Our findings suggest that drugs (excluding antivenom), ward charges, and nursing procedures are the highest contributors to the total indirect cost of managing snakebite. When antivenom is added to the valuation, the problem of managing snakebite in a local hospital setting suddenly becomes more complex. We also report that the median cost of treating snakebite was 2652 KES (~$26). According to a recently published survey, nearly half of Kenyan households earn less than 10,000 KES (~$97) per month, while 2% have no income at all ^12^. What this means is that, in the event of a snakebite, around 50% of all Kenyan households would need to spend approximately 25% of their monthly income treating the condition, and another 2% would have no means of paying for the treatment. Moreover, the minimum wage in Kenya is currently KES 13,572 (~$134) per month, which translates to about KES 452 (~$4) per day ^13^. Hence, for a person who misses work for 10 days due to hospitalization, the lost revenue would be about KES 4,520 (~$44). In essence, this person becomes poorer as they may not be able to buy food for their family, pay rent, or pay school fees for their children. Their productivity is therefore dented, which is bound to affect their socioeconomic standing. Moreover, the four individuals in this study who died from their injuries may also have an impact on cost, as these individuals will no longer be financially available for their families who are most likely to slide into poverty.

From our findings, the longer a snakebite victim was in the hospital, the more likely they were to incur higher costs. Additionally, we established that spending more than 10 days in the hospital was associated with a 62% increase in the total cost, relative to spending between one and five days in hospital. A length of stay of between six and 10 days was associated with a 32% increase in the total cost of treating the snakebite at the hospital.

The first guidelines for the prevention, diagnosis, and management of snakebite envenoming in Kenya were published in April 2019 ^6^. These guidelines provide details of how snakebite envenoming should be managed at the different levels of care in Kenya, including at the rural dispensary/health center, sub-county/district hospital, and at the referral hospital level ^6^. Based on our findings, there was no evidence that the 20-minute whole blood clotting test or any urine exam, considered as standard diagnostic tests in snakebite guidelines, were done in any of these facilities. Moreover, non-steroidal anti-inflammatory drugs (NSAIDs) such as diclofenac, ketorolac, aceclofenac, and ibuprofen were routinely given to victims of snakebite, despite their use being contraindicated in managing the disease. It was also evident that some of the bites had hematological effects, including slight/mild/minimal bleeding, severe/excessive bleeding, hyper-pigmentation at the site of the bite, ecchymosis, and erythema. NSAIDs interfere with the blood coagulation cascade and may potentiate hematological disturbances in the event of a snakebite, particularly those from snakes with hemotoxic venom ^6^. It is interesting to note that all the victims who died had received an NSAID (intramuscular diclofenac in particular) for snakebite-associated pain.

According to the clinical notes (particularly those that had something to do with the referral chain), the unavailability of antivenom was the main reason for referring victims from the smaller facilities to either the sub-county/district hospital or to the referral hospital. At the sub-county/district hospital level, conservative management of snakebite by use of blood transfusion or fresh frozen plasma (FFP) was often not possible as the blood/FFP was seldom available at the facilities. Furthermore, sub-county/district hospitals had to refer patients to JOOTRH for an array of reasons, including the unavailability of antivenom, the lack of the pre-requisite reagents to perform clinical and laboratory examinations, and the lack of ECG/radiography services, as well as a lack of snakebite management skills. 

At JOOTRH, antivenom was also not always available, and there were many cases of victims being asked to buy the antivenom from private pharmacies in the town of Kisumu. This was a challenge, especially for cases that occurred at night. Many of the private pharmacies in the area do not operate at night and most did not stock antivenom, further delaying treatment. Furthermore, based on our findings, there was no evidence that bacterial cultures were used to inform the choice of antibiotics for managing snakebite-associated infections. This observation is troubling considering the current state of antimicrobial resistance in developing countries including Kenya. The fact that very few victims were subjected to laboratory evaluation is also quite alarming. The reasons for this observation need to be elaborated.

Despite all the shortcomings in managing snakebite at JOOTRH, there were a few positives. First, based on the two cases of gangrene that were resolved successfully (after fasciotomy), it seems that the surgical team at the facility is well equipped to manage venomous snakebite-induced necrosis. Second, it was good to see that rehabilitative services were offered to some victims by the department of physiotherapy and occupational therapy. Third, the availability of psychological services to snakebite victims was also commendable. This was exemplified by the case of a 16-year-old female victim of snakebite who had a psychiatric episode secondary to the bite, who was discharged three days after presenting to JOOTRH having received antivenom, supportive care (IV fluids) and psychological counseling.

Kisumu East sub-county contributed at least four in every ten cases of snakebite at JOOTRH during the study period. The area has several wards including Central Kolwa, East Kajulu, West Kajulu, East Kolwa, and Manyatta wards ^14^. JOOTRH is also located in Kisumu East. We therefore posit that the numbers of patients presenting to the hospital from this region may be related to the proximity of the victims to the hospital.

There were more bites during the rainy season than the dry season, in agreement with other similar studies ^15, 16^. This observation may have something to do with the fact that snakes seek dry and safe shelter whenever heavy rains disrupt their habitats ^17^.

Our findings of more bites occurring in the rural areas, predominantly affecting the young people, mostly being inflicted on the lower limbs and occurring in the late evenings are consistent with the reports of other similar studies ^18– 22^.

The demographic that was most affected by snakebite included students and individuals partaking in outdoor activities. Moreover, most bites occurred while the victims were walking. In rural Africa, more often than not, students have to travel long distances to have access to education. We posit that these students may have been bitten in the evening hours as they walked back home from school.

The symptoms of snakebite envenoming and the description of the offending snakes are largely consistent with the type of snakes known to be in the area. These snakes are mostly of the Viperidae and Elapidae families ^6^ and include the puff adder ( *Bitis arietans*), gaboon viper ( *Bitis gabonica*), rhinoceros viper ( *Bitis nasicornis*), black mamba ( *Dendroaspis polylepis*), Jameson’s mamba ( *Dendroaspis jamesoni*), the eastern forest cobra ( *Naja subfulva*), and the gold tree cobra ( *Pseudohaje goldi*) ^6^.

More than half of all the victims did not attempt any pre-hospital first aid measures. A similar number reported to the hospital within six hours of having been bitten. When taken collectively, this may suggest that the locals recognize that snakebite is a medical emergency requiring urgent medical intervention. On the other hand, the lack of initiative by the victims or their proxies in attempting any pre-hospital measure may suggest that the population is not knowledgeable on the appropriate steps to take in the event of snakebite. Where some form of pre-hospital measure was used, the measure adopted cannot be considered as beneficial. Some of the measures are contraindicated and have been shown to do more harm than good ^23^. The use of tourniquets, incisions, suction, heat, ice, alcohol, and electric shock have all been reported to be counterproductive in snakebites ^23^. The jury may still be out on the role of herbal medicine in snakebites. Those that oppose the practice argue that seeking treatment from traditional herbal medicine practitioners often delays access to proper medical intervention and may result in complications ^24, 25^. In contrast, proponents of herbal medicine argue that the purpose of herbal medicine is not to replace antivenom, but serve an adjunctive role, particularly in managing local effects of envenomation such as necrosis, as has been reported by several authors ^26– 28^. The latter seems to be buoyed by the fact that some phytochemicals isolated from medicinal plants have shown some promising *in vitro* and *in vivo* neutralization capacity against phospholipase A _2_ and metalloproteases, which are enzymes associated with local tissue damage ^26– 29^.

It is difficult to see how other practices such as burning matchsticks at the site of the bite, the use of a cloth impregnated with charcoal, the application of petroleum jelly at the site of the bite, the use of potassium permanganate and povidone-iodine, and pouring paraffin at the site of the bite could mitigate against snakebite envenoming. There is a need for public health awareness programs aimed at dissuading such harmful practices from being advanced in the management of snakebite among this population.

### Limitations

The cost of medicines and health services in Kenya are yet to be standardized and as such the indirect costs we have provided are mere estimates and may be higher or lower in other hospital settings. Moreover, this study did not capture information on the victims of snakebite who may have died on their way to the hospital or those who sought treatment outside the hospital’s catchment area. Furthermore, owing to the retrospective nature of this study, it was not possible to capture information on other costs incurred by victims, such as the costs incurred in transporting the victims to the hospital. 

## Conclusions

Snakebite injuries contribute significantly to medical costs in the hospital setting. The longer snakebite victims stay in hospital, the higher the cost. Continuous medical education on the correct management of snakebites should be encouraged to minimize snakebite-related complications that may increase hospital stay and, consequently, the cost incurred by victims. Prospective work is needed to provide better estimates of the direct and indirect costs of snakebite injuries in the hospital setting.

## Data availability

### Underlying data

Figshare: Raw data on the study titled ‘management and cost of snakebite injuries at a teaching and referral hospital in Western Kenya. https://doi.org/10.6084/m9.figshare.9642005.v2 ^11^

This project contains the following underlying data:

- Snake bite injuries-working data -modified.xlsx (raw demographic, medical and cost data for each participant)

### Extended data

Figshare: Extended data on the study titled ‘management and cost of snakebite injuries at a teaching and referral hospital in Western Kenya’. https://doi.org/10.6084/m9.figshare.9204773.v5 ^10^

This project contains the following extended data:

- Figure S1: Ethical approval document from the ethical review committee of Jaramogi Oginga Odinga Teaching and Referral Hospital
- Table S1: A summary of the hospitals that referred victims of snakebite to JOOTRH during the study period
- Table S2: A summary of the utility of antivenom among victims of snakebite presenting to JOOTRH during the study period
- Table S3: A summary of the waivers received by some victims of snakebite at JOOTRH during the study period

Data are available under the terms of the Creative Commons Zero "No rights reserved" data waiver (CC0 1.0 Public domain dedication).
