# Supplementary material for: Management and cost of snakebite injuries at a teaching and referral hospital in Western Kenya
Source: F1000Res. 2019 Sep 4;8:1588. [Version 1] doi: 10.12688/f1000research.20268.1 (PMC6892383; doi:10.12688/f1000research.20268.1)
Supplement: Peer review history

# Peer review: Reviewer response for version 1

**DOI:** 10.5256/f1000research.22270.r55941

**Published:** 2019-12-05

**Authors:** Oommen Oommen

**Affiliations:** 1 Department of Computational Biology and Bioinformatics, University of Kerala, Thiruvananthapuram, Kerala, 695581, India

I have read the above manuscript with interest and found it to be a good survey followed by their conclusions. The manuscript is well written with the data available and help understand the lacunae in snake bite management in Kenya. The study included authentic data collected for 5 years from JOOTRH between 2011-2016. They have screened 127 medical records having full desired data and made the analysis.They followed international classification from medical archives to arrive at their conclusions. The snake bite still remains a neglected tropical disease in Kenya as is similar in other tropical countries. The fact that snake bite victims have to travel a long distance to avail antivenom is certainly a big draw back. Even in some cases, the antivenom is still not available in place like JOOTRH. The results of the present study should be made available to Health officials to take adequate measures for future snake bit management. Ideally local county hospitals should be empowered to reduce travel time and cost of treatment. Antivenom may be made available in referral hospitals to reduce, mortality and cost of treatment especially when the average monthly income of citizen is $44. The quality of antivenom also to be improved, probably with support from UN. It is welcome to take initial traditional and dependable herbal medicines and avail anitvenom from nearest county hospitals.The authors deserve credit for their commitment and concern for snake bite cases in Kenya.

 The manuscript is well written and the language is good. I recommend the manuscript for indexing in your journal and also with a request to implement the important findings of this study to reduce the snake bite deaths and sufferings of the survivors.

I confirm that I have read this submission and believe that I have an appropriate level of expertise to confirm that it is of an acceptable scientific standard.

# Peer review: Reviewer response for version 1

**DOI:** 10.5256/f1000research.22270.r56985

**Published:** 2019-12-03

**Authors:** Godpower Chinedu Michael

**Affiliations:** 1 Department of Family Medicine, Aminu Kano Teaching Hospital, Kano, Nigeria

**Comments**

 General comment:

- Thank you inviting me to review this manuscript. The title of this manuscript is apt and is of relevance in many resource-limited settings.

Specific comments:

- Introduction: this beautifully written.
- Methods: Here, I appreciate the description of the study setting and reason for the choice of the period to which medical charts were reviewed (in this case, 5 years). However, I will suggest a review of your definition of costs in the economic evaluation of snakebite injuries. Generally and broadly there are two types of costs:
   
  1. Direct cost: Refers to the resource consumed in providing health care interventions. It consist of:
    Direct medical cost: cost of hospital stay, outpatient visits, card/consultation fees, drugs, labs, surgery, physiotherapy, etc
    Direct non-medical cost: these resources supporting medical services such as transportation cost, caregiver costs, etc.
  2. Indirect cost: this refers to cost from production losses. These include cost due to incapacity to work, disability or premature death.
- If “complications including death” from snakebite were your outcome measures, then the nomenclature should be reworded to reflect these, instead of “direct cost” as it is presently.
- Data handling and statistical analysis: I struggled to understand what qualitative variables were analyzed through thematic analysis. Your study was of quantitative design; for instance, no focused group discussions were held. This area needs clarification or else the statement I am referring to should be deleted.
- The results may need to reflect the queries above.
- Conclusion: reasonably written.

I confirm that I have read this submission and believe that I have an appropriate level of expertise to confirm that it is of an acceptable scientific standard, however I have significant reservations, as outlined above.

# Peer review: Reviewer response for version 1

**DOI:** 10.5256/f1000research.22270.r55940

**Published:** 2019-11-21

**Authors:** Charles F.B. Nhachi

**Affiliations:** 1 Department of Clinical Pharmacology, College of Health Science, University of Zimbabwe, Harare, Zimbabwe

This is a very interesting and important subject to interrogate especially in the Sub Saharan region where snake bite is a significant public health concern even though it is a neglected one. The financial burden of snake bite injuries on the health sector is even more neglected despite the fact that it is significant. So this study is indeed a welcoming breath of fresh air so to speak.

 This work is acceptable for indexing with some minor corrections/revisions. While most of the current citations were referred to the paper can be enriched by citing work which has been carried out in the region, e.g. South Africa and Zimbabwe, to emphasise the commonality of the problem in the region.

 The authors could also add references from the WHO  “Guidelines for the Prevention and Clinical Management of Snakebites in Africa”. Although the guidelines are not very recent, they are still the prevailing ones.

 What is the age group of “child” according to the authors?

 What is the relevance of marital status to snakebite injuries?

 Circumstances of injury, “walking”, can this be explained further…walking where?

 What is “homemaking”?

 Perhaps the length of hospitalisation, the site of injury and the kind of snake can be associated to give a more vivid picture. Also the association of cause of death, snake type and injury can be elaborated.

 Snake identification can be confusing and difficult. A black snake is not necessarily a black mamba. A black mamba is in fact not black! It's black inside the mouth.

 What was the cause of death of the two who died after receiving antivenom? Was it due to reaction to the antivenom or late medical attention and what was the kind of injury?

I confirm that I have read this submission and believe that I have an appropriate level of expertise to confirm that it is of an acceptable scientific standard.

# Peer review: Reviewer response for version 1

**DOI:** 10.5256/f1000research.22270.r55305

**Published:** 2019-11-12

**Authors:** Benjamin Wachira

**Affiliations:** 1 Accident and Emergency Department, Aga Khan University Hospital (AKUH), Nairobi, Kenya

The article is well written and easy to follow.

 There is a need to classify all the 127 bite victims into the 3 categories Cytotoxic, Haematotoxic and Neurotoxic and any combinations of the same seen so it's clear how many patients had what symptoms.

 Subsequently, the patients should be followed down along these pathways so it's clear for each category. It's hard to tell if the 74 who did not get antivenom, did they have any symptoms? They may not have had toxic snake bites or were these excluded from the study...not clear.

 As all the data is available, we should be able to track the different patient categories through their hospital stays and also determine the outcomes for each of the categories.

I confirm that I have read this submission and believe that I have an appropriate level of expertise to confirm that it is of an acceptable scientific standard, however I have significant reservations, as outlined above.
